# Regulation of INSIG2 by microRNA-96

**DOI:** 10.1080/19768354.2017.1336483

**Published:** 2017-06-13

**Authors:** Youngah Jo, Ji-Young Cha, Young-Ah Moon

**Affiliations:** a Department of Molecular Genetics, University of Texas Southwestern Medical Center, Dallas, TX, USA; b Department of Biochemistry, Lee Gil Ya Cancer and Diabetes Institute, Gachon University, Incheon, South Korea; c Department of Molecular Medicine, Inha University School of Medicine, Incheon, South Korea

**Keywords:** Mir-96, insulin-induced gene, sterol regulatory element-binding protein

## Abstract

Mature forms of the microRNAs miR-96, -182, and -183 originate from a single genomic locus and have been shown to be elevated approximately 50-fold in the livers of sterol regulatory element-binding protein-1a and -2 (*SREBP-1a* and *-2*) transgenic mice. Our study attempted to identify the possible targets of these microRNAs using miRNA target prediction software. This revealed putative sites in insulin-induced genes (*INSIGs*). The 3′ untranslated region (UTR) of insulin-induced gene 1 (*INSIG1*) contained sites corresponding to miR-182, and -183, while the 3′ UTR of *INSIG2* featured an miR-96 site. Among these putative sites, only miR-96 demonstrated an inhibitory effect that was specific to the 3′ UTR of *INSIG2*. As INSIG proteins are the main components of SREBP cleavage complexes that act to release active SREBPs, we assessed the effects of miR-96 on INSIG and SREBP levels and activities. We found that miR-96 reduced the levels of INSIG2 in *INSIG1* knockout human fibroblasts, resulting in an increase in SREBP-1 and -2 nuclear forms and a subsequent increase in the abundance of the mRNA of their target genes. These results suggest that miR-96, an miRNA induced by SREBP-2 activation, regulates downstream targets of SREBPs and may increase the abundance of active SREBP.

## Introduction

Cholesterol and fatty acids are the main components of cell membranes and are primarily synthesized from acetyl-CoA. The enzymes responsible for *de novo* cholesterol and fatty acid synthesis are regulated by sterol regulatory element-binding proteins (SREBPs). Three isoforms of SREBPs have been identified in mammals, SREBP-1a, -1c, and -2. SREBP-1c and SREBP-2 are the most prevalent isoforms found in adult tissue and are produced as inactive precursors. These are typically found as integral membrane proteins in the endoplasmic reticulum (ER). SREBPs contain an N-terminal transcription factor region that is released in the Golgi apparatus by two proteases, site-1 (S1P) and site-2 protease (S2P). This cleaved region moves to the nucleus where it activates target genes by binding sterol response elements (SREs). SREBP-1c preferentially regulates genes involved in fatty acid and triglyceride synthesis, while SREBP-2 activates genes involved in cholesterol synthesis, low-density lipoprotein receptors, and PCSK9 (Brown & Goldstein 1997; Horton & Shimomura 1999; Horton et al. 2002).

N-terminal cleavage of these SREBPs is regulated by several accessory proteins, such as SREBP cleavage activating protein (SCAP), insulin-induced gene 1 (INSIG1), and INSIG2. In situations where there is sufficient ER cholesterol, INSIGs bind to SCAP and prevent the SCAP-SREBP complex from moving to the Golgi apparatus (Yabe et al. 2002; Yang et al. 2002). However, when cholesterol is low in the ER, SCAP undergoes a conformational change that results in dissociation from INSIG proteins. This enables SCAP-SREBP to be incorporated into COP-II-coated vesicles that move to the Golgi apparatus, where the N-terminal region of SREBP is released (Sun et al. 2005). Free INSIG1 that dissociates from SCAP undergoes rapid ubiquitin-mediated proteosomal degradation, while INSIG2 has a longer half-life and is not regulated by sterols (Gong et al. 2006; Lee et al. 2006).

The target genes of each SREBP isoform have been identified through the study of livers from three types of mice that either overexpress nSREBP-1a (TgSREBP-1a), overexpress nSREBP-2 (TgSREBP-2), or are liver-specific SCAP knockouts (KO) (*Scap^−/−^*) that have reduced expression for all SREBPs in liver (Shimano et al. 1996; Horton et al. 1998; Matsuda et al. 2001). In particular, genes that encode enzymes required for fatty acid and cholesterol synthesis were identified as the likely targets for SREBP-1 and -2, respectively (Horton et al. 2002; Horton et al. 2003). Additionally, SREBPs can also regulate non-coding RNAs. For example, a polycistronic microRNA (miRNA) locus that contains miR-96, -182, and -183 was identified as a non-coding RNA region that is directly activated by SREBP-2 (Jeon et al. 2013). In our study, we have identified a predicted binding site for miR-96 in the 3′ untranslated region (UTR) of *INSIG2* and determined its role in the regulation of INSIG2 protein and SREBPs.

## Experimental procedures

### RNA isolation and miRNA qPCR

C57BL/6J mice were fed a normal chow diet *ad libitum* until the start of the experiment (Teklad Mouse/Rat Diet 2018, Harlan Teklad Premier Laboratory Diets). One group of mice (*n* = 5) were fasted for 12 h and then their livers were collected and frozen (low insulin group). A second group (*n* = 5) was fed a high carbohydrate diet (MP Biomedicals, Cat. No. 960238) for 12 h after the initial 12 h fasting period (high insulin group). Livers from the mice described above and livers from Tg-albumin-Cre;*Scap^f/f^* mice (*n* = 5) (liver-specific *Scap* KO mice) were obtained from Dr. Jay Horton in University of Texas Southwestern Medical Center. All animal studies were approved by the IACUC of University of Texas Southwestern Medical Center. RNA was isolated from frozen livers as indicated in the manufacturer’s manual with minor modification. RNA was precipitated overnight in 70% isopropanol at –20°C. Reverse transcription reactions were performed using a TaqMan microRNA Reverse Transcription Kit (Life Technologies) and the quantity of each miRNA was measured using TaqMan microRNA Assays (Life Technologies). miR-96 levels were normalized to U6 RNA levels.

### Generation of pFOXO1, -INSIG1, and -INSIG2 clones

The 3′ UTR regions of *INSIG1*, *INSIG2*, and *FOXO1* were amplified using genomic DNA isolated from HepG2 cells and the primers FOXO1, 5′-TCTAGAGGGTTAGTGAGCAGGTTACACTTAA-3′ and 5′-GTCGACAGGTCCAAGGCTGTTCAATGGAGAT-3′; INSIG1A, 5′-TCTAGAAGATCGGGCTGACTGTACAAATGAC-3′ and 5′-GTCGACATTGTCTACACAAACTGCCACGGGA-3′; INSIG1B, 5′-TCTAGATCAGCAGAATGGAAGCTTAGAGGAA-3′ and 5′-GTCGACCTTAGTATGAATGTGAACCTCACTAG-3′; INSIG2, 5′-TCTAGATACTGCAATCTGTGATTGCTTCATC-3′ and 5′-GTCGACTCTGCTCATCACATATACTTCCAGT-3′. PCR products were digested using *Xba*I and *Sal*I and inserted into the *Xba*I and *Sal*I sites of a pmirGLO dual-luciferase plasmid (Promega). Resulting plasmids were designated pFOXO1, pINSIG1A, pINSIG1B, and pINSIG2. The putative binding sites of miR-183 in INSIG1A and miR-96 in INSIG2 were deleted from pINSIG1A and pINSIG2 plasmid using a Quick Change Lightning Multi Site Directed Mutagenesis Kit (Agilent) and 5′-CATGTGATTAAAACAAGTTTTCAAAGCCTTGAACTA-3′, and 5′-TGTATCACAATGTTAATGATATTGTTCCTGTCATG-3′ primers, respectively. The putative sites for miR-182 and -96 in FOXO1 were deleted from pFOXO1 using the primer 5′-AAATTTCATTACAATGAACTCACTACACCATATAAT-3′, and for miR-183 using primer 5′-CTGCTGTAGATAAGGACTTGGAAATTTCATTACAAT-3′.

### Luciferase assays using pmirGLO clones

CHO-K1 cells were obtained from the Korean Cell Line Bank (KCLB No. 10061) and cultured in Ham’s F-12 medium containing 10% fetal bovine serum and 1 × Antibiotic-Antimycotic (Life Technologies). Human *mir*VANA miRNA mimics were obtained from Life Technologies. These included hsa-miR-96 (assay ID: MC10422), hsa-miR-182 (assay ID: MC12369), hsa-miR-183 (assay ID: 12830), and negative control #1 (cat# 4464058). Cells were plated at a density of 2.5 × 10^3^ in 48-well plates on day 0. On day 2, pGLO plasmids (0.3 μg/well) and *mir*VANA miRNA mimics (0, 1, 3, 10, and 20 nM) were transfected into cells using DharmaFECT-Duo Transfection Reagent (GE Healthcare). Cell lysates were prepared in 1× lysis buffer (Promega). Firefly luciferase and *Renilla* luciferase activities were measured using a Dual-luciferase System (Promega). Firefly luciferase activity was normalized to *Renilla* luciferase activity.

### Transfection of miRNAs and analysis of protein and mRNA levels

*INSIG1* (TR4145, *INSIG1*-KO) or *INSIG 2* (TR4148, *INSIG2*-KO) KO human fibroblasts were kind gifts from Dr. Russell DeBose-Boyd at the University of Texas Southwestern Medical Center. On day 0, *INSIG1*-KO and *INSIG2*-KO cells were plated onto 100 mm dishes at densities of 5 × 10^5^ and 6 × 10^5^, respectively. On days 1 and 2, cells were transfected with 20 nM *mir*VANA miRNA mimics and 20 μL Lipofectamine RNAiMAX (Life Technologies) per plate. On day 3, cells were harvested and the membrane and nuclear proteins, and total RNA, were prepared as described previously (Jo, Sguigna et al. 2011). A 30 μg aliquot of protein from each sample was subjected to sodium dodecyl sulfate polyacrylamide gel electrophoresis and immunoblots using antibodies raised against INSIG1 (13F5), INSIG2 (17H1), SREBP-1 (20B12), SREBP-2 (22D5), SCAP, cAMP response element-binding protein, and calnexin (Jo, Lee et al. 2011; Moon et al. 2012; Jo et al. 2013; Rong et al. 2017). Total RNA was isolated using an RNA STAT solution and treated with DNaseI from a DNA-free kit (Ambion). cDNA was synthesized using a TaqMan Reverse Transcription Kit (Life Technologies). Real-time qPCR was performed using 2×SYBR Green PCR Master Mix (Life Technologies), as previously described (Liang et al. 2002).

## Results

### miR-96 levels were not regulated by insulin

**Figure 1.**
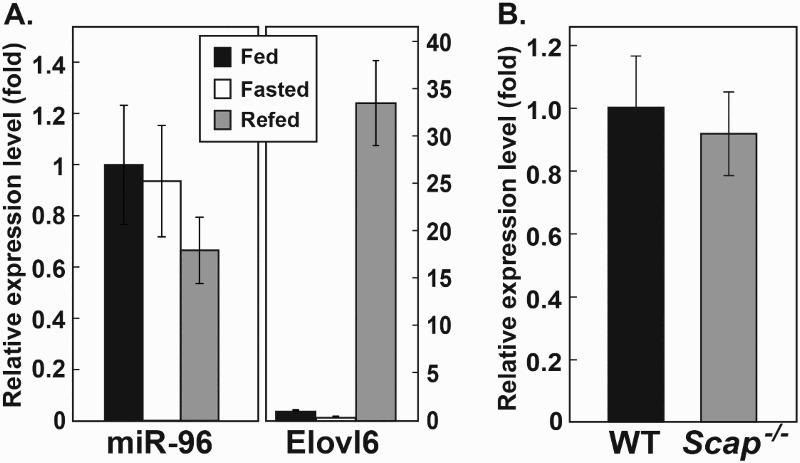
Mature miR-96 levels in the livers of mice. (A) Mature miR-96 levels were measured in the liver of mice fed on a chow diet *ad libitum*, fasted for 12 h (low insulin), or fed a high carbohydrate diet for 12 h after the 12 h fasting period (high insulin). The mRNA levels of *ELOVL6* were used to assess the response to each dietary condition. (B) Mature miR-96 levels were measured in the livers of WT mice and liver-specific *SCAP* knockout mice. miR-96 levels were normalized to the level of *U6* and *ELOVL6* mRNA levels were normalized to cyclophilin. The values obtained in WT mice were regarded as 1.0 and used to estimate relative expression in other groups. Values indicate the means ± S.E. (*n* = 5 mice). ELOVL6, elongation of very long chain fatty acid-like family member 6.

miR-96 was previously identified as a miRNA originating from the polycistronic miRNA locus that also contains miR-182 and -183. This miRNA has previously been shown to be highly elevated (approximately 50-fold) in the livers of TgSREBP-1a, and TgSREBP-2 transgenic mice when compared to wild-type (WT) (Jeon et al. 2013). To determine whether insulin also regulates miR-96, levels of the miRNA were measured in livers of mice fasted for 12 h (low insulin) and mice refed a high carbohydrate diet for 12 h after 12 h of fasting (high insulin). No significant changes were detected in the levels of mature miR-96 between the fasting and refeeding periods (Figure 1(A)). The basal expression level of miR-96 was extremely low in WT mice but there was no further reduction in miR-96 levels in *Scap^−/−^* mice compared to WT. This suggests that SREBPs are not critical to the maintenance of basal levels of miR-96 (Figure 1(B)). These results also indicate that short-term changes in insulin level do not play a significant role in regulating miR-96.

### The 3′ UTR of *INSIG2* is inhibited by miR-96

**Figure 2.**
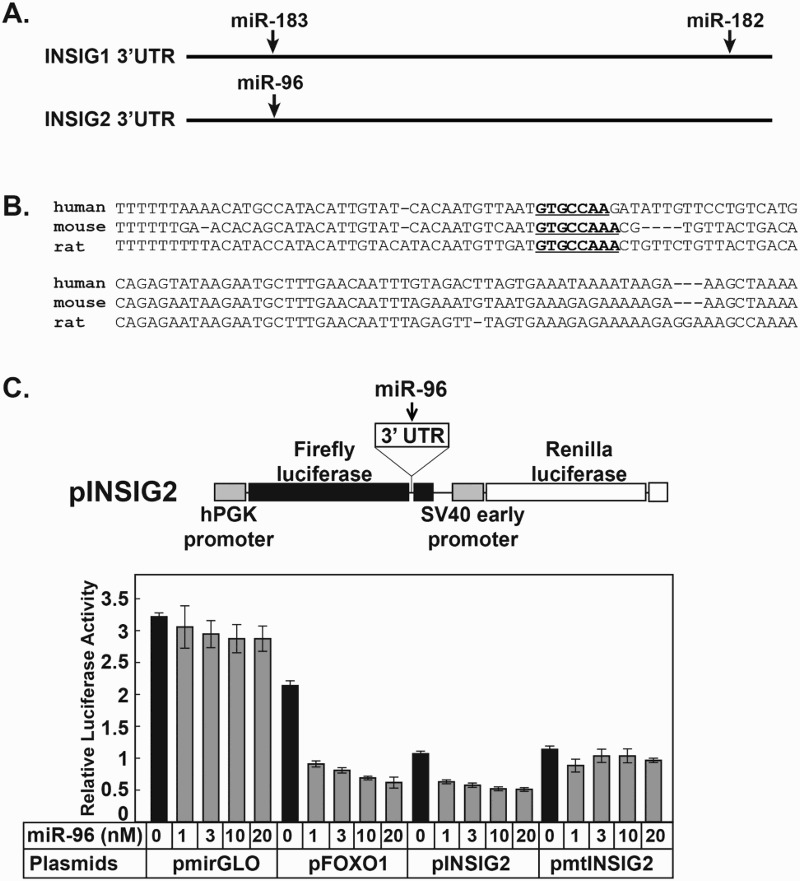
Inhibitory effects of miR-96 on the 3′ UTR of *INSIG2*. (A) Putative binding sites for miR-96, –182, and –183 were identified across the genome. The 3′ UTRs of *INSIG*s were predicted as possible targets. The predicted binding sites for each miR are shown. (B) The same putative miR-96 binding site was found in the 3′ UTR regions of human, mouse, and rat *INSIG2*. The binding sequence for miR-96 is shown in bold, underlined characters. (C) A region approximately 500 bp in length encompassing the 3′ UTR of human *INSIG2* containing the miR-96 binding site was cloned into a pmirGLO plasmid downstream of a firefly luciferase gene (pINSIG2). The same 3′ UTR region, but with the putative miR-96-binding site deleted was also cloned into a similar plasmid (pmtINSIG2). A plasmid containing the 3′ UTR of human *FOXO1* was used as a positive control. Plasmids were then co-transfected into CHO-K1 cells with miR-96 mimics. Cell extracts were prepared and the firefly and *Renilla* luciferase activities measured. Firefly luciferase activity was normalized to the *Renilla* luciferase activity. Values shown are means ± S.E. (*n* = 4 wells).

Targetscan and miRDB software were used to search for the target genes of miR-96, -182, and -183. This identified putative binding sites for miR-96 in the 3′ UTR of *INSIG1* and for miR-183 in the 3′ UTR of *INSIG2*. Furthermore, these sites were found to be conserved in the mouse and rat genomes (Figure 2(A)). Putative binding sites for miR-182 were also identified in the 3′ UTR of human and mouse *INSIG1* (Figure 2(A)). To determine whether the putative binding sites for miR-96, -182, and -183 in the 3′ UTRs of *INSIG1* and *INSIG2* were regulated by miRNAs, we cloned a 500 bp region from *INSIG1* and *INSIG2* containing these binding sites into a pmirGLO plasmid downstream of a firefly luciferase reporter gene. The 3′ UTR of *FOXO1* was used as a positive control (Guttilla & White 2009; Myatt et al. 2010). CHO-K1 cells were co-transfected with each plasmid and miR-96, -182, and -183 mimics. The firefly luciferase activity of each cell extract was then measured. This revealed that the luciferase activity of pFOXO1 was significantly inhibited by the presence of miR-96, -182, and -183. The luciferase activity of the pINSIG2 plasmid was inhibited by approximately 50% in cells transfected with miR-96, even when using concentrations as low as 1 nM. The inhibitory effects of miR-96 were abolished when the putative binding site for miR-96 was deleted from the 3′ UTR of *INSIG2* (Figure 2(C)). The luciferase activities of plasmids containing the 3′ UTR regions of *INSIG1* and *INSIG2* with the putative binding sites for miR-182 and miR-183 were unaffected by the addition of miR-182 or miR-183 mimics (data not shown). These results suggest that miR-96 may inhibit INSIG2 by binding to the predicted site we identified in the 3′ UTR of *INSIG2.* Finally, we confirmed that the miR-96 binding site found in the 3′ UTR of mouse *Insig2* behaved similarly.

### Endogenous INSIG2 is inhibited by miR-96

**Figure 3.**
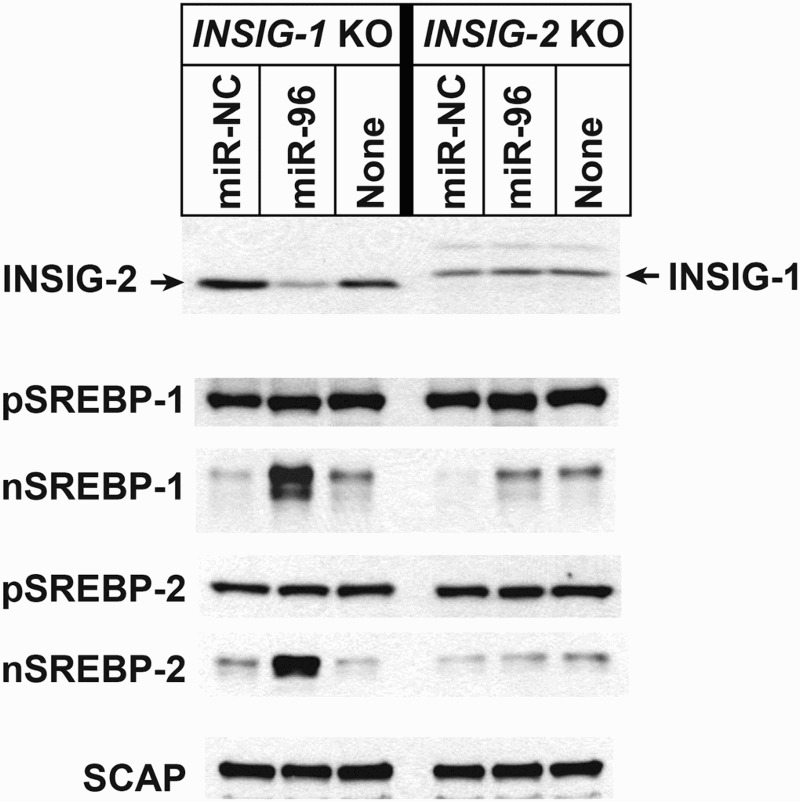
Immunoblots of INSIGs and SREBPs in human fibroblasts transfected with a miR-96 mimic. *INSIG1* (*INSIG-1* KO) or INSIG2 (*INSIG-2* KO) knockout human fibroblasts were transfected with a control miRNA (miR-NC) or a miR-96 mimic. Nuclear and membrane proteins were prepared from the cells and immunoblotting performed using the indicated antibodies. ‘None’ indicates cells without transfection.

**Figure 4.**
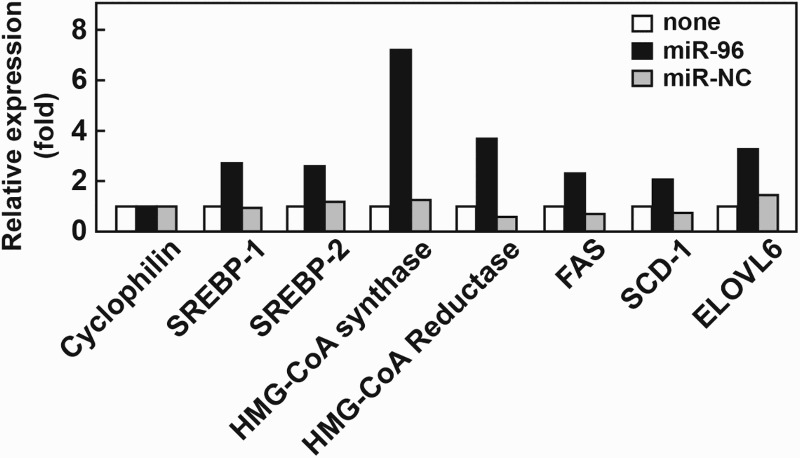
mRNA expression levels of SREBP target genes in human fibroblasts transfected with miR-96. Total RNA was prepared from cells transfected with either miR-NC or a miR-96 mimic. The expression levels of the target genes of SREBP-1 (*FAS*, *SCD-1*, and *ELOVL6*) and SREBP-2 (HMG-CoA synthase and HMG-CoA reductase) were measured. The expression levels in cells without transfection were regarded as 1.0 and levels in other groups determined relative to this group. Data were normalized to a cyclophilin endogenous control (*n* = 2). *FAS*, fatty acid synthase; *SCD-1*, stearoyl-CoA desaturase; *ELOVL6*, elongation of very long chain fatty acid-like family member 6.

To establish whether miR-96 can regulate endogenous INSIG1 or INSIG2 protein and affect SREBP processing in cells, human fibroblasts were transfected with miR-96 and the abundances of INSIG1 and INSIG2 were compared to cells transfected with a negative control miRNA (miR-NC) and non-transfected controls. Human fibroblasts in which *INSIG1* or *INSIG2* had individually been deleted (designated *INSIG1*-KO and *INSIG2*-KO) were used to assess the changes to each protein and any downstream effects more clearly. In *INSIG1*-KO cells, INSIG2 levels were significantly decreased in the presence of miR-96. The mature forms of SREBP-1 and SREBP-2 were also increased in the *INSIG1*-KO cells transfected with miR-96, suggesting an association with INSIG2 and miR-96. No changes were detected in the levels of SCAP (Figure 3). However, we found that adding miR-96 did not affect INSIG1 levels in *INSIG2*-KO cells and there was no subsequent effect on SREBP activation. Changes in the expression of the target genes of SREBP-1 and SREBP-2 were also assessed in *INSIG1*-KO cells transfected with miR-96 or miR-NC (Figure 3). These genes are primarily involved in the cholesterol synthesis pathway and include 3-hydroxy-3-methylglutaryl-coenzyme A (HMG-CoA) synthase and HMG-CoA reductase. We found that the transcript levels of these genes were increased fourfold to sixfold in *INSIG1*-KO cells exposed to miR-96. Additionally, transcripts for genes involved in the fatty acid synthesis pathway, such as *FAS*, *SCD-1*, and *ELOVL6*, were increased twofold to fourfold in cells transfected with miR-96, alongside an increase in SREBP activation (Figure 4). These results suggested that miR-96 can inhibit endogenous INSIG2 and consequently enhance SREBP processing, leading to increased expression of SREBP target genes.

## Discussion

The roles of SREBPs in the regulation of enzymes required for fatty acid and cholesterol metabolism have been extensively investigated using various transgenic and KO mice models. This detailed analysis has contributed to a better understanding of how lipid metabolism is regulated (Shimano et al. 1996; Shimano et al. 1997; Matsuda et al. 2001; Liang et al. 2002). Regulation of non-coding genes by SREBPs, particularly miRNAs, has also been investigated (Jeon et al. 2013). For example, SREBP-2 was found to induce transcription of miR-96, -182, and -183, from a single region. Our study has shown that miR-96 targets the 3′ UTR of *INSIG2*, reducing its expression and thereby accelerating SREBP processing. This results in an increase in the abundance of the nuclear forms of SREBP-1 and -2, both of which have further roles in SREBP regulation. Another miRNA from the same transcript, miR-182 was shown to increase SREBP levels by inhibiting *Fbxw7* (Jeon et al. 2013). We were unable to determine a role for miR-183 in our study and found no effect on INSIG or SREBP activation.

miR-96 and miR-183 both have an additional role in controlling FOXO1, a major transcription factor in the liver that regulates glucose production genes, such as glucose 6-phophatase and phosphoenolpyruvate carboxykinase. The FOXO1 3′ UTR was used as a positive control in our study of regulation by miR-96 and miR-183. Although FOXO1 does not directly regulate SREBPs, a reduction in FOXO1 may increase the flow of substrates into the lipid synthesis pathway. By reducing the flow of glucose 6-phosphate into glucose production, it may be more available for fatty acid and cholesterol synthesis. The expression and subsequent regulation of miRNAs transcribed together may be both a way to rapidly activate SREBPs and a method to increase fatty acid and cholesterol synthesis. In concert with other regulation methods, including transcriptional and post-translational mechanisms, our study reveals that miRNAs act to control SREBP levels and also play a role in directing metabolic flow.

## References

[CIT0001] Brown MS, Goldstein JL. 1997 The SREBP pathway: regulation of cholesterol metabolism by proteolysis of a membrane-bound transcription factor. Cell. 89:331–340. doi: 10.1016/S0092-8674(00)80213-5 9150132

[CIT0002] Gong Y, Lee JN, Lee PCW, Goldstein JL, Brown MS, Ye J. 2006 Sterol-regulated ubiquitination and degradation of insig-1 creates a convergent mechanism for feedback control of cholesterol synthesis and uptake. Cell Metab. 3:15–24. doi: 10.1016/j.cmet.2005.11.014 16399501

[CIT0003] Guttilla IK, White BA. 2009 Coordinate regulation of FOXO1 by miR-27a, miR-96, and miR-182 in breast cancer cells. J Biol Chem. 284:23204–23216. doi: 10.1074/jbc.M109.031427 19574223 PMC2749094

[CIT0004] Horton JD, Goldstein JL, Brown MS. 2002 SREBPs:activators of the complete program of cholesterol and fatty acid synthesis in the liver. J Clin Invest. 109:1125–1131. doi: 10.1172/JCI0215593 11994399 PMC150968

[CIT0005] Horton JD, Shah NA, Warrington JA, Anderson NN, Park SW, Brown MS, Goldstein JL. 2003 Combined analysis of oligonucleotide microarray data from transgenic and knockout mice identifies direct SREBP target genes. Proc Natl Acad Sci USA. 100:12027–12032. doi: 10.1073/pnas.1534923100 14512514 PMC218707

[CIT0006] Horton JD, Shimomura I. 1999 SREBPs: activators of cholesterol and fatty acid biosynthesis. Curr Opin Lipidol. 10:143–150. doi: 10.1097/00041433-199904000-00008 10327282

[CIT0007] Horton JD, Shimomura I, Brown MS, Hammer RE, Goldstein JL, Shimano H. 1998 Activation of cholesterol synthesis in preference to fatty acid synthesis in liver and adipose tissue of transgenic mice overproducing sterol regulatory element-binding protein-2. J Clin Invest. 101:2331–2339. doi: 10.1172/JCI2961 9616204 PMC508822

[CIT0008] Jeon T-I, Esquejo Ryan M, Roqueta-Rivera M, Phelan Peter E, Moon Y-A, Govindarajan Subramaniam S, Esau Christine C, Osborne Timothy F. 2013 An SREBP-responsive microRNA operon contributes to a regulatory loop for intracellular lipid homeostasis. Cell Metab. 18:51–61. doi: 10.1016/j.cmet.2013.06.010 23823476 PMC3740797

[CIT0009] Jo Y, Hartman IZ, DeBose-Boyd RA. 2013 Ancient ubiquitous protein-1 mediates sterol-induced ubiquitination of 3-hydroxy-3-methylglutaryl CoA reductase in lipid droplet–associated endoplasmic reticulum membranes. Mol Biol Cell. 24:169–183. doi: 10.1091/mbc.E12-07-0564 23223569 PMC3564538

[CIT0010] Jo Y, Lee PCW, Sguigna PV, DeBose-Boyd RA. 2011 Sterol-induced degradation of HMG CoA reductase depends on interplay of two Insigs and two ubiquitin ligases, gp78 and Trc8. Proc Natl Acad USA. 108:20503–20508. doi: 10.1073/pnas.1112831108 PMC3251157 22143767

[CIT0011] Jo Y, Sguigna PV, DeBose-Boyd RA. 2011 Membrane-associated ubiquitin ligase complex containing gp78 mediates sterol-accelerated degradation of 3-hydroxy-3-methylglutaryl-coenzyme A reductase. J Biol Chem. 286:15022–15031. doi: 10.1074/jbc.M110.211326 21343306 PMC3083207

[CIT0012] Lee JN, Gong Y, Zhang X, Ye J. 2006 Proteasomal degradation of ubiquitinated insig proteins is determined by serine residues flanking ubiquitinated lysines. Proc Natl Acad Sci USA. 103:4958–4963. doi: 10.1073/pnas.0600422103 16549805 PMC1405624

[CIT0013] Liang G, Yang J, Horton JD, Hammer RE, Goldstein JL, Brown MS. 2002 Diminished hepatic response to fasting/refeeding and LXR agonists in mice with selective deficiency of SREBP-1c. J Biol Chem. 277:9520–9528. doi: 10.1074/jbc.M111421200 11782483

[CIT0014] Matsuda M, Korn BS, Hammer RE, Moon YA, Komuro R, Horton JD, Goldstein JL, Brown MS, Shimomura I. 2001 SREBP cleavage-activating protein (SCAP) is required for increased lipid synthesis in liver induced by cholesterol deprivation and insulin elevation. Genes Dev. 15:1206–1216. doi: 10.1101/gad.891301 11358865 PMC313801

[CIT0015] Moon Y-A, Liang G, Xie X, Frank-Kamenetsky M, Fitzgerald K, Koteliansky V, Brown MS, Goldstein JL, Horton JD. 2012 The scap/SREBP pathway Is essential for developing diabetic fatty liver and carbohydrate-induced hypertriglyceridemia in animals. Cell Metab. 15:240–246. doi: 10.1016/j.cmet.2011.12.017 22326225 PMC3662050

[CIT0016] Myatt SS, Wang J, Monteiro LJ, Christian M, Ho K-K, Fusi L, Dina RE, Brosens JJ, Ghaem-Maghami S, Lam EW-F. 2010 Definition of microRNAs that repress expression of the tumor suppressor gene FOXO1 in endometrial cancer. Cancer Res. 70:367–377. doi: 10.1158/0008-5472.CAN-09-1891 20028871 PMC2880714

[CIT0017] Rong S, Cortés VA, Rashid S, Anderson NN, McDonald JG, Liang G, Moon Y-A, Hammer RE, Horton JD. 2017 Expression of SREBP-1c requires SREBP-2-mediated generation of a sterol ligand for LXR in livers of mice. eLife. 6:e25015. doi: 10.7554/eLife.25015 28244871 PMC5348127

[CIT0018] Shimano H, Horton JD, Hammer RE, Shimomura I, Brown MS, Goldstein JL. 1996 Overproduction of cholesterol and fatty acids causes massive liver enlargement in transgenic mice expressing truncated SREBP-1a. J Clin Invest. 98:1575–1584. doi: 10.1172/JCI118951 8833906 PMC507590

[CIT0019] Shimano H, Shimomura I, Hammer RE, Goldstein JL, Brown MS, Horton JD. 1997 Elevated levels of SREBP-2 and cholesterol synthesis in livers of mice homozygous for a targeted disruption of the SREBP-1 gene. J Clin Invest. 100:2115–2124. doi: 10.1172/JCI119746 9329978 PMC508404

[CIT0020] Sun L-P, Li L, Goldstein JL, Brown MS. 2005 Insig required for sterol-mediated inhibition of scap/SREBP binding to COPII proteins in vitro. J Biol Chem. 280:26483–26490. doi: 10.1074/jbc.M504041200 15899885

[CIT0021] Yabe D, Brown MS, Goldstein JL. 2002 Insig-2, a second endoplasmic reticulum protein that binds SCAP and blocks export of sterol regulatory element-binding proteins. Proc Natl Acad Sci USA. 99:12753–12758. doi: 10.1073/pnas.162488899 12242332 PMC130532

[CIT0022] Yang T, Espenshade PJ, Wright ME, Yabe D, Gong Y, Aebersold R, Goldstein JL, Brown MS. 2002 Crucial step in cholesterol homeostasis: sterols promote binding of SCAP to INSIG-1, a membrane protein that facilitates retention of SREBPs in ER. Cell. 110:489–500. doi: 10.1016/S0092-8674(02)00872-3 12202038

